# Mass Spectrometry of Collagen-Containing Allogeneic Human Bone Tissue Material

**DOI:** 10.3390/polym16131895

**Published:** 2024-07-02

**Authors:** Nikolay A. Ryabov, Larisa T. Volova, Denis G. Alekseev, Svetlana A. Kovaleva, Tatyana N. Medvedeva, Mikhail Yu. Vlasov

**Affiliations:** 1 Research Institute of Biotechnology “BioTech”, Samara State Medical University of the Ministry of Health of the Russian Federation, 443079 Samara, Russia; n.a.rjabov@samsmu.ru (N.A.R.); volovalt@yandex.ru (L.T.V.); m.yu.vlasov@samsmu.ru (M.Y.V.); 2 Core Shared Research Facility “Industrial Biotechnologies”, Federal Research Center “Fundamentals of Biotechnology” of the Russian Academy of Sciences, 117312 Moscow, Russia; s.kovaleva@fbras.ru

**Keywords:** bone tissue, allogeneic biomaterial, biopolymers, collagen, hydrogel, mass spectrometry, biofabrication, 3D bioprinting

## Abstract

The current paper highlights the active development of tissue engineering in the field of the biofabrication of living tissue analogues through 3D-bioprinting technology. The implementation of the latter is impossible without important products such as bioinks and their basic components, namely, hydrogels. In this regard, tissue engineers are searching for biomaterials to produce hydrogels with specified properties both in terms of their physical, mechanical and chemical properties and in terms of local biological effects following implantation into an organism. One of such effects is the provision of the optimal conditions for physiological reparative regeneration by the structural components that form the basis of the biomaterial. Therefore, qualitative assessment of the composition of the protein component of a biomaterial is a significant task in tissue engineering and bioprinting. It is important for predicting the behaviour of printed constructs in terms of their gradual resorption followed by tissue regeneration due to the formation of a new extracellular matrix. One of the most promising natural biomaterials with significant potential in the production of hydrogels and the bioinks based on them is the polymer collagen of allogeneic origin, which plays an important role in maintaining the structural and biological integrity of the extracellular matrix, as well as in the morphogenesis and cellular metabolism of tissues, giving them the required mechanical and biochemical properties. In tissue engineering, collagen is widely used as a basic biomaterial because of its availability, biocompatibility and facile combination with other materials. This manuscript presents the main results of a mass spectrometry analysis (proteomic assay) of the lyophilized hydrogel produced from the registered Lyoplast^®^ bioimplant (allogeneic human bone tissue), which is promising in the field of biotechnology. Proteomic assays of the investigated lyophilized hydrogel sample showed the presence of structural proteins (six major collagen fibers of types I, II, IV, IX, XXVII, XXVIII were identified), extracellular matrix proteins, and mRNA-stabilizing proteins, which participate in the regulation of transcription, as well as inducer proteins that mediate the activation of regeneration, including the level of circadian rhythm. The research results offer a new perspective and indicate the significant potential of the lyophilized hydrogels as an effective alternative to synthetic and xenogeneic materials in regenerative medicine, particularly in the field of biotechnology, acting as a matrix and cell-containing component of bioinks for 3D bioprinting.

## 1. Introduction

Biofabricated products are prepared using materials which have a significant potential for human tissue replacement by acting as the cells’ carrier matrices. An area of clinical medicine with widespread application of biofabricated products is orthopaedics; which requires such materials to repair bone defects that are unrecoverable with conventional treatment. To solve this serious problem, numerous studies have been conducted that propose new biomaterials that can be resorbed after implantation and replaced by the patient’s tissue [1,2,3,4]. Biopolymers of allogeneic origin are an example of such materials [5,6]. They have a number of advantageous features, including biocompatibility, low immunogenicity, biomimeticity, biodegradability and, significantly, the ability to provide a beneficial environment with structural support for cells and their adhesion [7,8,9,10]. These properties allow biomaterials to perform their specific functions for the required period of time without any damage to the organism, acting as bioimplants. When placed into the body, such a bioimplant undergoes gradual biodegradation mediated by the microenvironment’s components and is eventually replaced with the native tissue. There are recent studies reporting the successful application of allogeneic donor biomaterials for the reconstruction of the complex defects of bone and of the surrounding soft tissues of the extremities [1,2].

Such biomaterials can form a construct serving as a container and/or attractant for cells, and initially as a source of their nutrition, as well as containing auxiliary components (factors of angiogenesis, cell proliferation and differentiation). Subsequently, the cells of the construct ensure the formation of a new extracellular matrix (ECM) in the area of the resorbed biomaterial [11].

**Figure 1:**
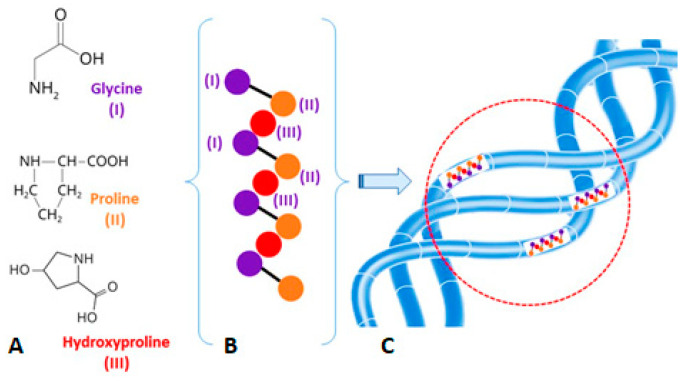
(**A**) Chemical formulas of the parent amino acids; (**B**) Chain of amino acids; (**C**) Triple helix structure of rope-like collagen molecule. Amino acid chains in the strands of the molecule are highlighted with a red circle.

One of the most promising biopolymers, which is widely used in the field of biofabrication, is collagen, a connective tissue protein that has a specific triple-spiral tertiary structure (Figure 1). Collagen is critical for the ECM’s structural and biological integrity, as well as cell metabolism and tissue morphogenesis, conferring the required mechanical and biochemical properties on the latter [12,13,14].

As of today, about 28 types of collagen are known; however, collagen type I is the most common in the ECM, as it composes up to 90% of total protein in the connective tissue, particularly in tendons and bones. Consequently, collagen type I is one of the most sought-after materials in the field of biotechnology [14,15].

Collagen has significant potential in tissue engineering as a matrix material for the restoration of bone defects due to its high availability, biocompatibility, combinability with other materials, hydrophilic properties, low antigenic activity, biodegradability, high porosity of the formed constructs, etc. [14,16]. When implanted in the body, collagen stimulates reparative processes by promoting the synthesis of intrinsic collagen [17]. Regenerative medicine uses xenogeneic (animal-derived), allogeneic and autologous (human-derived donor and intrinsic, respectively) collagens.

Bioimplants containing xenogeneic collagen have a number of disadvantages, and the main one is the risk of the recipient’s immune response followed by possible rejection. Bioimplants with allogeneic collagen, in turn, currently attract greater attention as they have a number of benefits vs. the xenogeneic ones. Particularly, they possess the lower risk of immune response and rejection, a greater ability to induce the transformation of the stem cells to progenitors in the surrounding tissues, and the ability to provide mechanical support to the cellular component. This allows allogeneic biomaterials to be considered the “gold standard” of regenerative medicine. Schmitt T. et al. used human-derived collagen in the concentration of 6 mg/mL as a core material for producing bioinks, which allowed the obtaining of 3D-bioprinted constructs with higher shape precision and better cell viability vs. xenogeneic collagen-based bioinks [15].

Currently, a large number of study methods are used to analyse the protein components of biomaterials. Of these, mass spectrometry is the most sensitive, discoverable and versatile proteomic tool, which ensures assessment of the protein structure, modifications and interactions in tissues or cell populations [18,19,20,21]. Mass spectrometry has been used to analyse human connective tissue proteins as well as to predict pathologies, including cardiovascular diseases [22,23] and malignancies [24]. The method has also been applied to diagnose Alzheimer’s disease, and within the comparative studies of alternations in the composition and organization of normal and damaged tissue pairs in the development of cardiovascular, oncological diseases, fibrosis and musculoskeletal pathology [25]. So, the method allows for the identification of ECM proteins that have not been previously isolated, and would serve as diagnostic or predictive biomarkers or new therapeutic targets [23]. This suggests the effectiveness of mass spectrometry in different areas of personalized medicine. 

The method may also be suitable for tissue engineering, e.g., to analyse the protein composition of the biopolymers that have been used in 3D-bioprinting as a core part of bioinks. The latter contain living cells while biopolymers in hydrogel form act as an analogue of ECM. Such bioinks are used to create constructs similar to native tissues using 3D-bioprinting technologies. Since protein components from allogeneic biomaterials serve as raw materials for tissue biofabrication, analysis of the former is an actual and demanding task.

The goal of this research was to evaluate the protein’s composition by mass spectrometry of the freeze-dried hydrogel produced from Lyoplast^®^ bioimplant represented by allogeneic human bone tissue.

## 2. Materials and Methods

### 2.1. Characteristics of Sources and Materials

**Figure 2:**
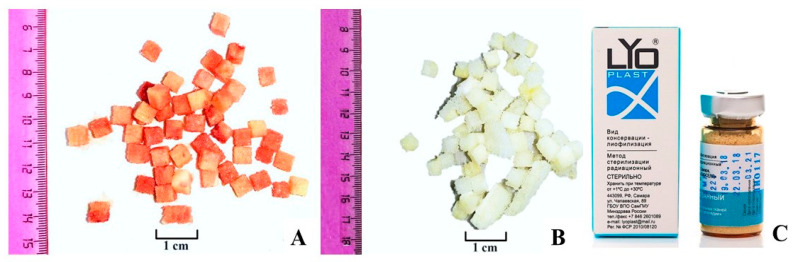
Bone tissue samples: (**A**)—Source material; (**B**)—Processed material; (**C**)—Hermetic sterile container Lyoplast^®^.

The Lyoplast^®^ bioimplant is a registered medical product represented by lyophilized and demineralized human bone tissue. Manufacturing is realized according to proprietary technology in the Samara tissue bank of the Research Institute of Biotechnology “BioTech”, Samara State Medical University (RF patent No. 2366173 of 05/15/2008; certificate of conformity ISO 13485:2016 [26], reg. No. RU CMS-RU.PT02.00115; certificate ISO 9001:2015 [27], reg. No. TIC 15 100 159171). The allogeneic bone tissue (postmortem material) was purified from blood, lipids and bone marrow stroma, then lyophilized, demineralized, sterilized and finally packed in a sealed sterile container. This technology preserves a number of organic components, scaffold proteins and bone tissue ECM proteins, including regeneration inducers and inhibitors [5,28,29]. (Figure 2). 

For this, bone samples underwent compulsory low-frequency ultrasonic action using a TTC Sapphire ultrasonic bath (Sapphire LLC, Moscow, Russia) at 24–40 kHz. Lyophilization of the material (vacuum drying by sublimation) was performed using an ALPHA2-4LSC sublimation unit (Martin Christ Gefriertrocknungsanlagen GmbH, Osterode am Harz, Germany). Demineralization of human bone tissue was carried out in a 2.4 M hydrochloric acid solution. The sealed lyophilized product was then sterilized with gamma rays using a certified GU-200 M gamma radiation sterilizer (NIIP Joint-Stock Company, Zhukovskiy, Russia). The residual content of lipids in the bioimplant was estimated using an SF-56 spectrophotometer (Lomo-Spektr, St. Petersburg, Russia); the humidity of the product was determined using a thermogravimetric infrared moisture meter MA-150 (Sartorius, Göttingen, Germany).

### 2.2. Studying Object

**Figure 3:**
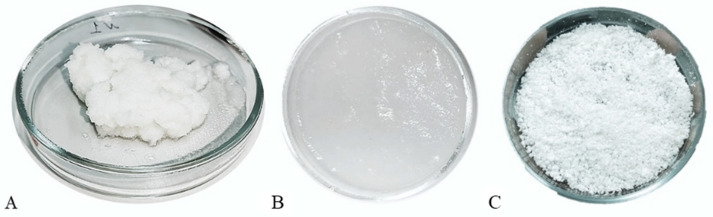
Hydrogel produced by the Lyoplast^®^ bioimplant: (**A**)—the primary purified material; (**B**)—hydrogel; (**C**)—freeze-dried hydrogel.

A freeze-dried hydrogel was produced from a Lyoplast^®^ bioimplant (lyophilized and demineralized human bone tissue) by chemical extraction of the protein complex with 30% acetic acid followed by neutralization of the hydrogel with 1 M sodium hydroxide solution. The hydrogel was dialyzed against distilled water for 24 h with twice water replacement and Spectra/Por^®^ 1 dialysis tubing MWCO 6000-8000 (SERVA, Heidelberg, Germany). To stabilize the hydrogel acidity, phosphate buffered saline with pH 7.4, 10×, diaGene (Diagene GmbH, Reinach, Switzerland) was added. Then the hydrogel was freeze-dried by vacuum drying using an AK 2-55 freeze drier (LyoGene, St. Petersburg, Russia). The freeze drying was performed in four steps: 5.00 mb, 0.50 mb, 0.30 mb, and 0.20 mb. (Figure 3). 

### 2.3. Sample Preparation

**Figure 4:**
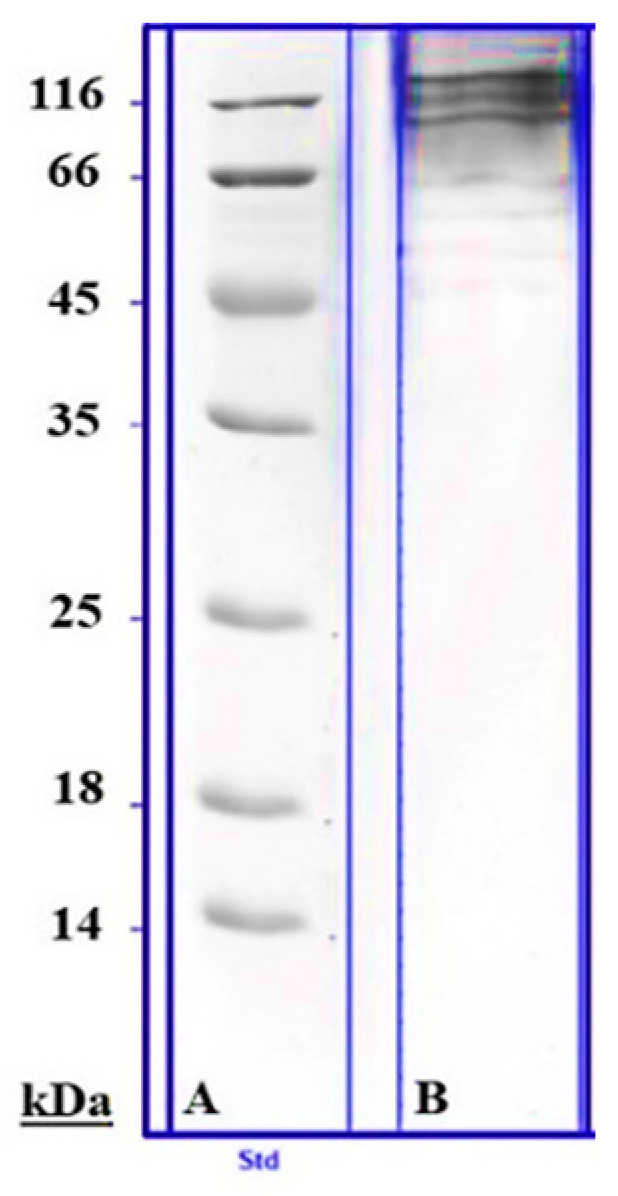
Protein separation by 10% SDS-PAGE. (**A**)—protein markers with known molecular weights; (**B**)—a sample of freeze-dried hydrogel.

The sample of freeze-dried hydrogel (hereinafter—sample) was heated (100 °C, 5 min) in the medium containing 2% sodium dodecyl sulfate (SDS) and 5% mercaptoethanol, then centrifuged with MiniSpin^®^ mini-centrifuge (Eppendorf, Hamburg, Germany) and separated by the electrophoresis in 10% polyacrylamide gel with SDS in Mini-PROTEAN Tetra cell (Bio-Rad, Berkeley, CA, USA). After electrophoresis, the gel was stained with Coomassie G250 and dissected into fragments in accordance with visible fractions (Figure 4) [30]. To identify the proteins, the separated fractions were excised from the gel, washed with 1:1 solution of ammonium hydrocarbonate and acetonitrile at 50 °C, dehydrated in 100% acetonitrile (ACN), and digested in the gel using trypsin (Trypsin Gold, Promega, Madison, WI, USA). For this, trypsin was resuspended at 1 µg/µL in 50 mM acetic acid and then diluted in 40 mM ammonium bicarbonate/10% acetonitrile to 20 µg/mL. The gel slices were preincubated for rehydration in a minimal volume (10–20 µL) of the trypsin solution at 24 °C for 1 h. The reaction was stopped with 0.1% trifluoroacetic acid; the peptides were isolated from the gel using an ultrasonic bath TTC Sapphire (Sapphire LTD, Moscow, Russia) and separated by ultra-high performance liquid chromatography (UPLC) using the Elute system (Bruker Daltonik GmbH, Bremen, Germany).

### 2.4. Mass Spectrometry (Proteomic Assay)

Ultra-high performance liquid chromatography–mass spectrometry (UPLC-MS) was performed using Impact II high resolution quadrupole time-of-flight mass spectrometer with the Apollo II Electrospray ionization source and the Elute UPLC system (Bruker Daltonik GmbH, Bremen, Germany) with Waters Acquity HSS T3 Column, 100Å, 1.8 µm, 2.1 × 100 mm reverse-phase column (Waters Technologies LTD, Dublin, Ireland) under the following conditions: injection volume of 50 µL, flow rate of 0.25 mL/min, gradient elution of 5–60% B for 30 min, then up to 95% B for 3 min (A: 0.1% formic acid in water, B: 0.1% formic acid in acetonitrile), column temperature of 30 °C, post-column flow separation of 1:20, electrospray ionization in positive ion mode, capillary potential of 4.5 kV, nebulizer gas: nitrogen, 1.0 bar, drying gas: nitrogen, 5.0 L/min, 200 °C, m/z scanning range 100–2200, full spectrum scanning frequency 2 Hz. Second order spectra automatic registration mode (activation by collision) with dynamic registration rate 2–8 Hz, cycle length 3.5 s, preferred charge states 2–6, collision gas: nitrogen, dynamic collision energy (23 eV at m/z 300, 65 eV at m/z 1300), automated internal calibration using sodium trufluoroacetate solution. Spectra processing and protein identification were performed in BioPharma Compass 3.1.1 (Bruker Daltonik GmBH, Bremen, Germany) and Mascot 2.8.1 (Matrix Science, London, UK). 

**Figure 5:**
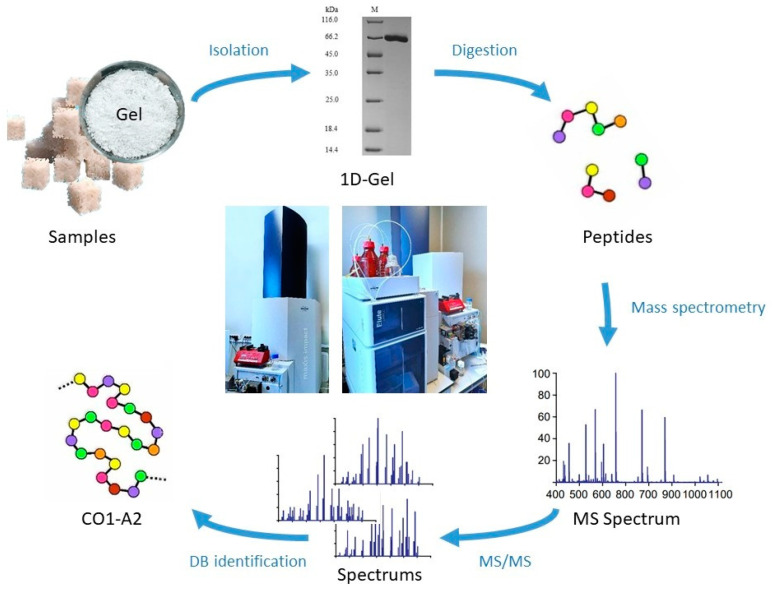
Scheme of the research with mass spectrometry (proteomic assay).

Figure 5 presents the scheme of the mass spectrometric (proteomic) assay of freeze-dried hydrogel produced by the Lyoplast^®^ bioimplant (allogeneic human bone tissue).

## 3. Results and Discussion

Proteins are high-molecular natural polymers consisting of amino acid residues connected by peptide bonds. They are the main constituent of living organisms and the molecular basis of life processes. Proteins play an important role in most essential biological processes and functions. They are versatile and perform different functions in the organism acting as catalysts, transport molecules, mechanical support elements and immune defence agents; also, they control cell growth and differentiation [30].

The structure of a protein determines its interaction with other molecules in the organism, and so defines its function. Any change in a protein at any structural level can affect its functionality [30]. 

The complex composition of different protein groups, which determine structural integrity and physiological functions, constitutes the extracellular matrix of connective tissues. The main function of the extracellular matrix is to give tissues specific mechanical and biochemical properties. The most common extracellular matrix proteins, which are the basic structural element of all connective tissues, belong to the collagen group. The ability of certain collagens to form networks and their anchoring function may contribute to the formation of scaffolds that promote tissue repair or regeneration [30].

Therefore, the study of the expression and function of different collagens, as well as comprehension of their molecular structure, biosynthesis, assembly and metabolism are important for understanding various biochemical processes at the stages of creating tissue-engineered constructs during 3D bioprinting of organs and tissues [13,14,15,16,17]. 

Mass spectrometry (proteomic assay) of the protein composition of the sample, represented by freeze-dried hydrogel produced by the Lyoplast^®^ bioimplant (allogeneic human bone tissue) detected the presence of six main collagen types:

- fibril-forming collagen type I;
- cartilaginous tissue-specific collagen type II;
- collagen type IV, the main structural component of basal membranes;
- collagen type IX, a hyaline cartilage component;
- collagen type XXVII, the protein essential for cartilage calcification and cartilage-bone transformation;
- collagen type XXVIII, cell-binding protein.

Furthermore, collagen membrane receptors, integrins (α10/β1), were identified, suggesting the participation of the collagen components in the initiation of signalling and enzymatic cascades in the cell populations growing on the collagen carriers. Collagen also binds the cells while promoting their adhesion by the integration of collagen bundles, the main component of the ECM [5].

The analysis of the test sample further revealed the following proteins: Integrin alpha-10 (ITGA10), Spectrin beta chain non-erythrocytic 2 (SPTBN2), Neuroblast differentiation-associated protein (AHNAK), Cryptochrome-1 (CRY1), Alanine-tRNA-ligase (AARS2), Jumonji protein (JARID2), ELAV-like protein 3 (ELAVL3), Bifunctional peptidase and arginyl hydroxylase JMJD5 (KDM8), Zinc finger protein 394 (ZNF394), Zinc finger protein 267 (ZNF267), Zinc finger protein 585 A (ZNF585 A), and a number of other proteins (Table 1).

The test sample is found to contain the compounds (Spectrin beta chain, non-erythrocytic 2 and Neuroblast differentiation-associated protein) critical for the cell membrane formation as well as nervous system cell proliferation and differentiation, i.e., the formation and innervation of new native tissues [5,39,40].

The presence of Cryptochrome-1, a transcription repressor and the main component of the circadian clock, may also be noteworthy. The circadian clock regulates various physiological processes by generation of about 24 h circadian rhythms of gene expression, which turn into metabolic and behavioural rhythms. By regulating gene and protein expression, circadian rhythms allow the organism to achieve time-dependent homeostasis with the environment at the molecular level. Transcription and translation of the clock main components (CLOCK, NPAS2, BMAL1, BMAL2, PER1, PER2, PER3, CRY1, and CRY2) are critical for the rhythm generation [33,41,42,43,44]. These proteins provide optimal conditions for the bioprinted constructs’ resorption with the formation of a new native ECM, as well as ensure the synthesis of the antioxidant and detoxifying enzymes that protect the cells.

The transcription/translation feedback loop (TTFL) forms the core mechanism of the molecular circadian clock. CLOCK transcription factors, namely NPAS2 and BMAL1/BMAL2, form the positive part of the feedback loop, operate as a heterodimer, and activate transcription of the CLOCK main genes involved in key metabolic processes and containing E-box elements (5′-CACGTG-3′) in their promoters. The CLOCK main genes and transcription repressors PER1/2/3 and CRY1/2 form the feedback loop negative part and interact with CLOCK(NPAS2)-BMAL1(BMAL2) heterodimer while suppressing its activity and thereby downregulating their own expression. CLOCK(NPAS2)-BMAL1(BMAL2) heterodimer also activates NR1D1/2 and RORA/B/G nuclear receptors, which form a second feedback loop and activate/suppress BMAL1 transcription, respectively. CLOCK(NPAS2)-BMAL1(BMAL2) can also downregulate target circadian gene expression in cooperation with HDAC1 and HDAG2 through histone deacetylation [33,45,54,55,56]. The presence of CLOCK(NPAS2)-BMAL1(BMAL2) in the freeze-dried hydrogel produced by the Lyoplast^®^ bioimplant (allogeneic human bone tissue) is important since such proteins regulate a broad spectrum of physiological functions, including cell metabolism. When such a hydrogel acts as a cell carrier, being a part of a bioink for 3D-bioprinting, CLOCK(NPAS2)-BMAL1(BMAL2) modulates the process of a new intrinsic ECM formation by cells.

The sample is also found to contain the proteins that catalyse amino acid activation (aminoacylation / tRNA charging) which, in particular, serve as a basis for the expression of synthetic functions of biological materials such as Lyoplast^®^ bioimplant (allogeneic human bone tissue) and the hydrogel produced from it. Thus, Alanine-tRNA ligase catalyzes charging of tRNA with alanine in a two-step reaction: activating alanine by adenosine triphosphate (ATP) to form alanine-adenosine monophosphate (Ala-AMP) at the first step, and transferring alanine to the tRNA acceptor site at the second one [45,46].

The sample also contains a histone methyltransferase complex regulator (Jumonji protein), which is critical for normal embryogenesis including heart, neural tube development and haematopoiesis. It acts as an additional subunit of PRC2 (Polycomb repressive complex 2) that mediates trimethylation of H3K27 histone on the chromatin, as well as binds DNA and promotes attraction of PRC2 to the target genes in the embryonal stem cells, playing a crucial part in the histogenesis, mainly at the embryonal stage [47,48,49]. Such proteins allow the maintenance of the normal physiological processes that occur when implanted biomaterials (e.g., Lyoplast^®^ and its derivatives) are replaced by native tissues.

ELAV-like protein 3 has also been identified in the sample. It binds to adenylate-uridylate-rich elements (AREs) of target mRNA sequences, including VEGF mRNA, and also contributes to mRNA stabilization [33,50]. ELAV-like protein 3 protein participates in the downregulation of neuronal stem cell proliferative activity and in the upregulation of the neuroblasts’ differentiation into the neurons. This protein also suppresses CDKN1A protein expression maintaining the proliferative state of the cells [33,57]. The presence of such a protein in the sample suggests the applicability of Lyoplast^®^ bioimplants and their derivatives in the field of biofabrication and bioprinting.

The crucial functions of the sample in the context of its chemical composition include its ability to regulate transcription. We have identified JMJD5 bifunctional peptidase and arginyl hydroxylase, which acts both as an endopeptidase and 2-oxoglutarate-dependent monooxygenase. The endopeptidase cleaves N-terminal histone fragments at the carboxy side of methylated arginine or lysine residues to form reduced nucleosomes, which can trigger the transcription elongation. The bifunctional enzyme also preferentially recognizes and cleaves monomethylated and dimethylated arginine residues in H2, H3 and H4 histones, regulates circadian gene expression in the liver, and inhibits the CLOCK-BMAL1 heterodimeric transcription activator in a catalytically independent manner [51,52]. The presence of the JMJD5 bifunctional peptidase in the Lyoplast^®^ bioimplants and their derivatives ensures the regulation of the synthetic processes during the postprocessing stage of bioprinting, providing the optimal conditions for the maturation of printed constructs. 

Zinc finger proteins (Zfp 267, Zfp 394, Zfp 585A) were also detected in the sample. Zinc fingers are the protein motifs, which interact with DNA, RNA, other proteins, or small molecules [53]. Zfp structure represents a small motif stabilized by one or two zinc ions, which are bound to the amino acid residues within the protein using coordinate bonds. A zinc finger generally comprises about 20 amino acids, and the zinc ion binds two histidines and two cysteines. Zfp proteins are known to be a background for such an analysis and are widespread in proteomic samples. Meantime, it is acknowledged that the main group of zinc finger proteins consists of DNA-binding transcription factors. The concordance of these data to the provided characteristics of the transcription and translation regulators, transcription activator, and repressor genes suggests the participation of zinc finger proteins in the metabolic processes during the resorption of printed and implanted constructs with followed histogenesis of the new intrinsic tissues.

Our research presents the results of the mass spectroscopy of the protein composition of freeze-dried hydrogel produced by a Lyoplast^®^ bioimplant (allogeneic human bone tissue) and provides a description of characteristic features of the components identified. Known physiologic functions of the latter offer an action mechanism of the allogeneic sources mentioned above and used in the experiment. They provide optimal settings, including circadian regulation, for resorption processes in bioprinted and implanted constructs with parallel synthesis of new intrinsic ECM (i.e., histogenesis). The synthesis of a new ECM is promoted by conductive properties of the construct biomaterial and is carried out by cells contained herein; if it is a hybrid cell-tissue product, or by mesenchymal stem cells (MSCs) attracted to the construct from the bloodstream or surrounding tissues. The study results also indicate that the designed method for the sample preparation allows the latter to provide the physiological and typical bone tissue process of reparative regeneration. This is a significant advantage of the Lyoplast^®^ bioimplant (allogeneic human bone tissue) and derived hydrogel, which is used as a bioink matrix and cell container in 3D-bioprinting.

## 4. Conclusions

The sample of the freeze-dried hydrogel, as well as its source, namely the bioimplant Lyoplast^®^ (allogeneic human bone tissue), contains intact structure collagen proteins. Accordingly, the derived hydrogel may also be considered as a polypotent material with a marked spectrum of transcription and translation regulatory activities. When implanting the Lyoplast^®^ products (e.g., bioimplants of allogeneic human tissue and derived hydrogels) in a human organism, processes of physiological reparative regeneration are observed. The fine mechanisms of the latter are caused by the protein compositions of Lyoplast^®^ products most similar to the ECM of native tissue. As such, the hydrogel, which comprises the described proteins, is thought to be a promising material for 3D-bioprinting in pure form or as basic part of a bioink, acting as a matrix and cell container. The hydrogel-bioprinted tissue-engineered constructs may be applied in regenerative medicine to repair the damage caused by diseases and injuries of connective, particularly supporting, tissue.

## Figures and Tables

**Table 1:** Proteins identified in freeze-dried hydrogel produced by the Lyoplast^®^ bioimplant (allogeneic human bone tissue) and their basic functions.

Ref. No.	Protein	Encoding Gene	Functions	Molecular Weight, kDa
1.	Collagen alpha-2(I) chain	COL1A2	Participates in collagen fibril arrangement, provides a structural component of the ECM [12,13,31,32]	129.2
2.	Collagen alpha-1(I) chain	COL1A1	Participates in collagen fibril arrangement, provides a structural component of the ECM [14,16,33]	138.9
3.	Collagen alpha-1(II) chain	COL2A1	Structural component of the ECM, confers tensile properties, binds metal ions, proteoglycans and platelet-derived growth factor, provides protein homodimerization activity [12,13,33]	141.7
4.	Collagen alpha-2(IV) chain	COL4A2	Structural component of basal membranes. Has both anti-angiogenic and anti-tumor activities. Inhibits endothelial cell proliferation and migration, decreases mitochondrial membrane potential and induces apoptosis [14,33]	167.4
5.	Collagen alpha-2(IX) chain	COL9A2	Structural component of hyaline cartilage, the main structural component of basal membranes [33]	65.1
6	Collagen alpha-1(XXVII) chain	COL27A1	Participates in the cartilage calcification and cartilage-bone transformation [33]	186.8
7.	Collagen alpha-1(XXVIII) chain	COL28A1	Participates in the cell binding (a cell-binding protein) [33]	116.6
8.	Integrin alpha-10	ITGA10	Collagen’s membrane receptor, integral transmembrane glycoprotein consisting of non-covalently bound alpha and beta chains. Participates in the cell adhesion as well as in the cell surface-mediated signaling. Differential pattern of integrin’s expression is mediated by growth and differentiation factors and may indicate participation of integrin in bone and cartilage metabolism [33,34,35,36,37,38]	127.5
9.	Spectrin beta chain, non-erythrocytic 2 (SPTBN2) and Neuroblast differentiation-associated protein (AHNAK)	SPTBN2 AHNAK	Cell membrane formation. Neurogenesis (proliferation and differentiation of nervous system cells) [5,33,39,40].	271.2
10.	Cryptochrome-1	CRY1	Transcription repressor, the main component of circadian clock. Transcription and translation of the main clock components (CLOCK, NPAS2, BMAL1, BMAL2, PER1, PER2, PER3, CRY1, and CRY2) [33,41,42,43,44]	66.4
11.	Alanine-tRNA ligase, mitochondrial	AARS2	Catalyst of amino acid activation (aminoacylation/tRNA charging) [33,45,46]	107.6
12.	Jumonji protein	JARID2	Regulator of histone-methyltransferase complexes. Participates in the stem cell differentiation and normal embryogenesis including heart, neural tube development and haematopoiesis [33,47,48,49]	138.3
13.	ELAV-like protein 3	ELAVL3	RNA-binding protein, stabilizes mRNA. Participates in the cell differentiation and nervous system development [33,50]	39.5
14.	Bifunctional peptidase (KDM8) and arginyl hydroxylase (JMJD5)	KDM8 JMJD5	Cleaves peptide bonds via hydrolysis reactions [33,51,52]	47.2
15.	Zinc finger protein 394, Zinc finger protein 267, Zinc finger protein 585 A	ZNF394 ZNF267 ZNF585 A	DNA-binding transcription factors [33,53]	64.2

## Data Availability

The data are provided upon request due to restrictions, for example, in the field of confidentiality and ethics.

## List of Abbreviations

AARS2	Alanine-tRNA-ligase
ACN	Acetonitrile
AHNAK	Neuroblast differentiation-associated protein
Ala-AMP	Alanine-adenosine monophosphate
AREs	Adenylate-uridylate-rich elements
ATP	Adenosine triphosphate
BMAL1/2	Brain and muscle arnt-like 1/2, or Arntl
CDKN1A	Cyclin Dependent Kinase Inhibitor 1A
CLOCK	Clock Circadian Regulator
COL1A1	Collagen alpha-1(I) chain
COL1A2	Collagen alpha-2(I) chain
COL27A1	Collagen alpha-1(XXVII) chain
COL28A1	Collagen alpha-1(XXVIII) chain
COL2A1	Collagen alpha-1(II) chain
COL4A2	Collagen alpha-2(IV) chain
COL9A2	Collagen alpha-2(IX) chain
CRY1	Cryptochrome-1
CRY1 (2)	Cryptochrome Circadian Regulator 1 (2)
ECM	Extracellular Matrix
ELAVL3	ELAV Like RNA Binding Protein 3
ITGA10	Integrin alpha-10
JARID2	Jumonji protein
JMJD5	Jumonji-C (JmjC) domain-containing protein 5
KDM8	Lysine Demethylase 8
MSCs	Mesenchymal stem cells
NPAS2	Neuronal PAS Domain Protein 2
NR1D1	Nuclear Receptor Subfamily 1 Group D Member 1
PER1/2/3	Period Circadian Regulator 1/2/3
PRC2	Polycomb repressive complex 2
RORA/B/G	Related Orphan Receptor A/B/G
SDS	sodium dodecyl sulfate
SDS-PAGE	sodium dodecyl sulfate polyacrylamide gel
SPTBN2	Spectrin beta chain non-erythrocytic 2
TTFL	Transcription/translation feedback loop
UPLC	ultra-high performance liquid chromatography
UPLC-MS	Ultra-high performance liquid chromatography—mass spectrometry
VEGF	Vascular endothelial growth factor
ZNF267	Zinc finger protein 267
ZNF394	Zinc finger protein 394
ZNF585 A	Zinc finger protein 585 A
H1/2/3/4	Histones 1/2/3/4
